# Spatial transcriptomics mapping of immune cell and TGFβ signalling pathway heterogeneity in testicular germ cell tumours

**DOI:** 10.1111/andr.70100

**Published:** 2025-07-22

**Authors:** Sarah C. Moody, Daniela Fietz, Benedict Nathaniel, Mark Frydenberg, Ben Tran, Hans‐Christian Schuppe, Kate L. Loveland

**Affiliations:** ^1^ Centre for Reproductive Health Hudson Institute of Medical Research Clayton Victoria Australia; ^2^ Department of Molecular and Translational Sciences, School of Clinical Sciences Monash University Clayton Victoria Australia; ^3^ Institute of Veterinary Anatomy, Histology and Embryology Justus‐Liebig University Giessen Giessen Germany; ^4^ Department of Surgery FMNHS, Monash University Melbourne Victoria Australia; ^5^ Department of Urology, Cabrini Institute Cabrini Health Melbourne Victoria Australia; ^6^ Department of Medical Oncology Peter MacCallum Cancer Centre Melbourne Victoria Australia; ^7^ Division of Personalised Medicine Walter and Eliza Hall Institute Parkville Victoria Australia; ^8^ Department of Urology, Pediatric Urology and Andrology Justus Liebig University Giessen Giessen Germany

**Keywords:** cancer immunobiology, interferon gamma, NanoString, testis cancer, TGFβ signalling pathway, tumour heterogeneity

## Abstract

**Background:**

Testicular germ cell tumours (TGCTs) are amongst the most common malignancies in young men, and their incidence is increasing worldwide. Tissue heterogeneity hampers efforts to understand how TGCT precursors (termed germ cell neoplasia in situ; GCNIS) emerge and progress, restricting elucidation of new strategies for diagnosis and management.

**Objectives:**

This study reports the use of spatial transcriptomic analysis in TGCT tissue sections.

**Materials and methods:**

Over 90 regions of interest (ROIs) were identified in sections from four TGCT patients’ samples, three with non‐seminoma, one seminoma. Transcripts in each ROI were sequenced and examined using the NanoString GeoMx spatial whole transcriptomics workflow, the values normalised and analysed using Degust RNA‐Seq and Ingenuity Pathway Analysis software.

**Results:**

The distribution and expression of functional markers in specific cell types was used to map individual tumours, GCNIS, and tumour‐adjacent regions. Significant heterogeneity in TGCTs and surrounding areas is documented between patients and across different regions in the same tumour. Immune cell‐related transcripts encoding macrophage, T cell, B cell, natural killer cell, dendritic cells and neutrophil subsets were identified as contributing substantially to tumour heterogeneity. Assessment of ROIs containing GCNIS and areas immediately adjacent from two individual non‐seminoma tumour samples identified the TGFβ family as contributing to upstream regulation of transcripts in both patients; known activin A target genes were differentially expressed between the GCNIS and microenvironment ROIs. In addition, two discrete tumour areas within the seminoma sample displayed distinct transcript profiles, one featuring higher levels of immune cell‐related transcripts, and the other TGFβ superfamily transcripts.

**Conclusion:**

These findings highlight aspects of the complex challenge faced while seeking therapeutic targets to enable tumour spread restriction. However, these outcomes reinforce knowledge that TGFβ family members can influence seminoma fate and provide new evidence of their potential contribution to the transition of GCNIS cells into tumours.

## 1 INTRODUCTION

The application of spatial transcriptomics to investigate testicular germ cell tumours (TGCTs) offers a unique opportunity to investigate cell populations in situ, including the complex immune cell milieu, the tumour microenvironment, region to region variations, and cell–cell signalling mechanisms. This technology has not been widely employed to compare differing TGCT subtypes, and this study illustrates how this approach may ultimately be applied to interrogate tumour status and impute aetiology following surgical excision of the testis from an affected patient.

The incidence of TGCTs is increasing worldwide, and they are among the most common tumours affecting young men; in the United States, testis cancer cases have increased from 12 to 14.3 cases per 100,000 (aged 20–39) between 2000 and 2017, while the incidence in Australia has increased from 7.3 to 9.9 cases per 100,000 (aged 30–39) between 2000 and 2024.^1^, ^2^, ^3^ In 2020, there were over 74,000 cases of testis cancer diagnosed globally and over 9000 deaths recorded.^4^ Despite a high cure rate for these tumours, lifelong burdens include heightened risk of cardiovascular disease, hypogonadism, sexual dysfunction, infertility and anxiety, with worse outcomes in men treated with chemotherapy and/or radiation.^5^, ^6^, ^7^, ^8^ Following detection of a testicular lump, serum markers such as α‐fetoprotein (AFP), lactate dehydrogenase (LDH) and β‐human chorionic gonadotropin (β‐hCG) are utilised to support the TGCT diagnosis, however, these are not specific to any TGCT subtype and normal levels do not exclude the presence of disease.^8^, ^9^, ^10^ Serum‐containing microRNAs are emerging as a key biomarker of TGCTs, with promising data indicating they are sensitive indicators of residual disease and valuable for long‐term monitoring. However, they are also not reliable indicators of tumour subtype or disease progression, and therefore diagnosis often occurs after orchiectomy.^11^, ^12^, ^13^ Along with the absence of apparent major predisposition genes,^14^ it is evident that other tools are required for early detection and tumour subtype‐specific diagnoses, in order to tailor therapeutics to the individual. The lack of fundamental knowledge about the aetiology of TGCT tumours is a major barrier.

TGCTs account for 95% of all testis cancer cases, arising due to a failure of foetal germ cell differentiation that leads to the formation of germ cell neoplasia in situ (GCNIS).^15^, ^16^ It is not known how latent GCNIS cells persist in the immature testis and then transform into homogeneous seminoma, heterogeneous non‐seminoma tumours, or mixed germ cell tumours. Because this change is made apparent after puberty, the hormonal cues governing testicular somatic cell maturation are regarded as integral to these outcomes.^17^, ^18^ Seminomas account for approximately 60% of TGCTs; similar to the precursor GCNIS, they express early germ cell markers including NANOG, OCT4, KIT, SOX17, PRAME and PDPN.^15^, ^19^, ^20^, ^21^ The heterogeneous non‐seminomas arise when GCNIS have differentiated into embryonal carcinoma, cells which can in turn differentiate into yolk sac, teratoma and choriocarcinoma tumours. Non‐seminomas, including embryonal carcinomas, are more prone to metastatic activity, and some retain pluripotency markers including OCT4, while embryonal carcinomas express SOX2, a transcription factor typically repressed in human primordial germ cells.^22^, ^23^

The progression of germ cells into GCNIS that transform into TGCT is considered to involve genetic and epigenetic changes, with an environmental component likely.^15^, ^24^ In addition to the gain of chromosome 12p,^25^ which appears to confer pluripotency and invasiveness, somatic mutations in *KIT*, *NRAS* and *KRAS* genes and DNA hypomethylation are common features of seminomas.^26^, ^27^ Non‐seminoma tumours containing embryonal carcinoma cells have a higher incidence of metastasis and exhibit moderate to strong levels of DNA 5mC methylation, associated with hypermethylation.^28^ The combined exposure of embryonal carcinoma cells to WNTs, transforming growth factor beta (TGFβ) and fibroblast growth factor (FGF) signalling stimuli has been shown to propel transformation of these cells into yolk sac tumour cells,^29^ an outcome which highlights the potential for the local microenvironment to drive GCNIS fate.

A feature of the many TGCTs is the presence of immune cells infiltrates, in contrast to the normal adult testis, where tissue resident macrophages are the most abundant immune cell type, and few T and no B cells are present.^16^, ^30^, ^31^, ^32^ Macrophages have been identified in all TGCT subtypes, including in GCNIS‐only areas. Testes containing GCNIS may have both T cells and B cells, with T cells more abundant.^30^, ^32^, ^33^ Clusters of B cells, T cells and dendritic cells (DCs) are documented in seminomas, while proportionately fewer T cells and higher numbers of DCs are identified in embryonal carcinomas when compared with seminoma tumours.^30^ The identification of both T regulatory and T follicular helper cells with contrasting suppressive and activating functions in primary tumours illustrates an observed TGCT tumour microenvironment complexity.^30^, ^34^ The significance of the differences between tumour types and regions within an individual tumour is not well understood, and understanding how individual immune cell types contribute to the microenvironment that controls GCNIS and tumour cell fate remains an important challenge that may inform clinical management.

Model cell lines have proven valuable for investigating processes that influence tumour cell behaviours. The two that are most commonly employed are the seminoma‐derived TCam‐2 line, which has many features of seminoma cells and gonocytes, and the testicular embryonal carcinoma‐derived NTera‐2 line that serves as a model of non‐seminoma tumour subtypes.^35^, ^36^ TCam‐2 cells have been utilised to investigate the role of immune cells; their co‐culture with peripheral blood mononuclear cells (PBMCs) increased cytokine mRNA production by TCam‐2 cells, indicating that interactions between both cell types may alter, and ultimately determine, the seminoma microenvironment.^37^ Phenotypic plasticity of TCam‐2 cells was observed after they were xenografted into the flank of nude mice; bone morphogenetic protein (BMP) pathway inhibition and the induction of *SOX2* were crucial for their subsequent transformation into EC‐like cells.^23^, ^38^, ^39^ These are examples of how the local signalling milieu may contribute to TGCT fate, including transformation of seminomas into the more malignant embryonal carcinoma/non‐seminoma tumours.

Knowledge of which molecules and signalling pathways regulate TGCT formation and progression is needed for the development of new diagnostics and to enable identification of therapeutic targets. However, the intra‐ and inter‐patient heterogeneity of tumours remains a significant obstacle. In this study, we employ spatial transcriptomics to examine three patient tumour samples containing non‐seminomas, and one containing seminoma, with the latter displaying two histologically distinct regions. By mapping the spatial distribution of these tumours and the diverse somatic cell types they contain, including immune cell populations, we demonstrate the potential for spatial transcriptomics to identify particular cell signalling pathways of relevance to the tumour niche and ultimately TGCT fate.

## 2 MATERIALS AND METHODS

### 2.1 Tissue samples

Fresh testis (tumour areas and macroscopically normal tissue adjacent to the tumour) was obtained from four patients undergoing orchidectomy following informed, written consent. Ethics were approved by Melbourne Health Human Research Ethics committee (HREC/15/MH/354), Cabrini HREC (12‐23‐01‐17) and Monash University HREC (RES‐20‐0000‐098C). Pathology reports were provided by TissuPath Pathology or Peter MacCallum Pathology for each patient (NonSem1–3, Sem) as summarised in Table 1. Tissue provided was identified as either primary tumour, or adjacent tissue (macroscopically normal) at the time of collection, then transported in ice‐cold phosphate‐buffered saline (PBS) or Hanks' balanced salt solution (HBSS). Each piece was halved into ‘side A’ and ‘side B’, then cut into segments. Some portions were fixed in 4% paraformaldehyde (PFA) in PBS (Alfa Aesar), then processed using standard ethanol and paraffin‐embedding procedures. Tissue sections were cut at 4 µm for standard immunostaining protocols, and 5 µm for the NanoString staining protocol. The remaining tissue underwent different processing protocols for other experiments.

**TABLE 1:** Summary of patient pathology reports.

Patient ID	Tumour type	Stage^a^	Reported pathology at collection
NonSem1	Non‐seminoma malignant mixed germ cell tumour	**pT1**	85% teratoma, 10% embryonal carcinoma and 5% yolk sac tumour. Small numbers of syncytiotrophoblastic cells present. Adjacent tissue shows background atrophy.
NonSem2	Non‐seminoma malignant mixed germ cell tumour	**pT1**	50% embryonal carcinoma, 30% yolk sac tumour and 20% teratoma. Necrosis present. Adjacent tissue shows patchy atrophy.
NonSem3	Non‐seminoma mixed germ cell tumour	**pT2**	90% embryonal carcinoma, 5% seminoma and 5% yolk sac tumour. germ cell neoplasia in situ (GCNIS) present
Sem	Seminoma	**pT1b**	100% seminoma. GCNIS present Testicular parenchymal atrophy

^a^ Pathology staging AJCC 8th edition.

### 2.2 Haematoxylin and eosin staining

Haematoxylin and eosin (H&E) staining was performed by staff at the Monash Histology Platform. Briefly, sections on slides were baked at 60°C for 20 min, then dewaxed in xylene, followed by ethanol rehydration. After rinsing in tap water, slides were incubated in Harris's Haematoxylin (Amber Scientific) for 7 min, followed by wash in tap water. Slides were dipped for 1 s in 0.5% acid alcohol, rinsed in tap water for 1 min and then placed in Scott's tap water for 1 min. After rinsing in tap water, slides were incubated with 1% alcoholic eosin (Amber Scientific) for 7 min. Sections were dehydrated in ethanol, cleared in xylene and coverslips applied with distyrene, plasticiser and xylene (DPX) mounting media. Imaging was performed using the Aperio Scanscope AT Turbo (Monash Histology Platform).

### 2.3 Immunohistochemistry

All steps were performed at room temperature unless otherwise stated, and all water wash steps performed using MilliQ water and standard methods, including 5‐min washes unless otherwise stated. Briefly, paraffin‐embedded sections on Superfrost Plus slides underwent dewaxing in histosol (Grale Scientific), followed by rehydration into water through a graded ethanol series (100%, 95%, 70% v/v). Antigen retrieval was performed using Tris–EDTA (10 mM Tris, 1 mM EDTA, pH 9) with 0.05% Triton‐X‐100 in a microwave. Slides were dipped in water after cooling, then treated with 3% hydrogen peroxide (Merck) for 5 min, rinsed in water, then washed (3×) in Tris‐buffered saline (TBS). After blocking in 3% bovine serum albumin (BSA; Sigma) for 30 min, sections were incubated overnight at 4°C with primary antibodies (anti‐OCT4, Santa Cruz, sc‐5279; anti‐CD68, DAKO, M0876), diluted at 1:100 and 1:1000 respectively in 0.5% BSA in TBS. A negative control section received no primary antibody. The following day, slides were washed three times in TBS, then sections were incubated with secondary antibody (Rabbit anti‐Mouse IgG Biotinylated, DAKO, E0354; 1:500 dilution for 1 h). After TBS washes (3×), sections were incubated for 30 min with Vectastain ABC reagent (PK‐6100). Sections were developed using 3,3'diaminobenzidine (DAB) (DAKO, K3468), washed in water, and counterstained with Harris’ Haematoxylin (Sigma). Slides were dehydrated in an ethanol gradient (70%, 95%, 100% v/v), placed in histosol, then mounted under coverslips with DPX (Sigma). Imaging was performed using the Aperio Scanscope AT Turbo (Monash Histology Platform).

### 2.4 NanoString GeoMX (staining, scanning, ROI selection)

**FIGURE 1:**
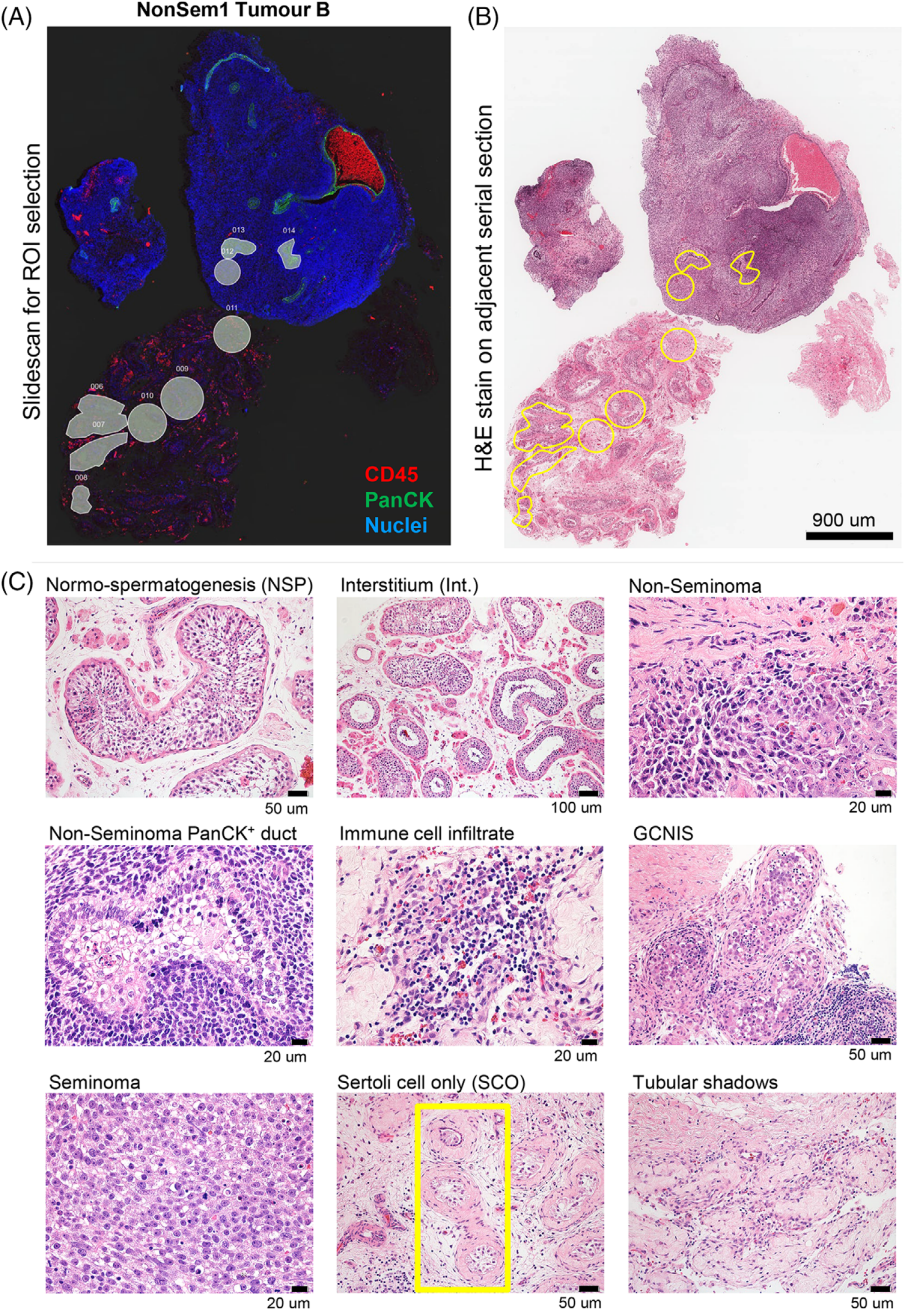
Region of interest (ROI) selection and annotation. (A) Immunofluorescence staining to detect immune cells (CD45), epithelial tumour cells (pan‐cytokeratin; PanCK) and nuclei. ROIs were selected on the Digital Spatial Profiler software using these images. (B) Haematoxylin and eosin (H&E) staining was performed on serial adjacent sections to aid with ROI selection and subsequent histological annotation. (C) High magnification images of representative ROIs from the H&E‐stained tissue. Yellow outlines mimicking the ROI outline on the fluorescent images.

PFA‐fixed, paraffin‐embedded testis tissue sections cut at 5 µm onto SuperFrost Plus slides were provided to the Peter MacCallum Centre for Advanced Histology and Microscopy within 2 weeks of sectioning. Slides and tissue sections are summarised in Table 2. Tissue preparation was performed according to the NanoString GeoMx NGS Manual Slide Preparation guidelines (NanoString) using the GeoMx slide Prep Kit, GeoMx Morphology Kit and GeoMx Whole Transcriptome Atlas Probe mix according to manufacturer's instructions. Briefly, slides were baked, deparaffinised and rehydrated. Antigen retrieval using Tris–EDTA was performed followed by incubation with Proteinase K to expose RNA targets. RNA probes were hybridised overnight at 37°C. Probes were washed off, and tissue sections blocked for 30 min. Morphology markers for pan‐cytokeratin (PanCK; endothelial cells) and CD45 (pan immune cell marker) and a DNA stain were applied and incubated for 1 h. Slides were washed, coverslipped and fluorescent scanning of tissue sections uploaded into the Digital Spatial Profiler (DSP) software. Alongside the immunofluorescence staining, H&E stains of adjacent serial sections were used to visualise the histology and aid in region of interest (ROI) selection (Figure 1A and Supporting Information Figures 1,3–6). Ninety‐five ROIs across the four patient tissue samples were initially selected on the DSP, with 92 analysed following quality control (QC; Supporting Information Figure 1). The nuclei count for each ROI ranged from 118 to 1133 nuclei, with an average of 384. Following ROI selection, RNA probes were photo‐UV‐cleaved and collected into a 96 well plate for sequencing.

**TABLE 2:** Slide layout of patient samples provided for spatial transcriptomics.

Section #	Patient ID	Tissue (adjacent or tumour)
Slide 1, section 1	NonSem1	Tumour A
Slide 1, section 2	NonSem1	Tumour B
Slide 1, section 3	NonSem2	Adjacent A Sections appear histologically normal (Supporting Information Figure 4)
Slide 2, section 1	NonSem2	Adjacent B Sections appear histologically normal (Supporting Information Figure 4)
Slide 2, section 2	NonSem2	Tumour
Slide 2, section 3	NonSem3	Adjacent A Tissue contained germ cell neoplasia in situ (GCNIS) (Supporting Information Figures 2 and 5)
Slide 3, section 1	NonSem3	Adjacent B Tissue contained GCNIS (Supporting Information Figures 2 and 5)
Slide 3, section 2	NonSem3	Tumour A
Slide 3, section 3	NonSem3	Tumour B
Slide 4, section 1	Sem	Adjacent Tubular atrophy present (Supporting Information Figure 6)
Slide 4, section 2	Sem	Tumour A
Slide 4, section 2	Sem	Tumour B

Brief descriptions of adjacent tissue histology are provided and can be observed (Supporting Information Figures 2 and 4–6).

### 2.5 Sequencing, data QC and normalisation

Sequencing of photo‐cleaved RNA oligo probes was performed at the Peter MacCallum Cancer Centre Molecular Genomics Core. Sequencing was performed using the NovaSeq NGS instrument. Initial evaluation of sequencing data determined that all ROIs had a strong signal, with the Quartile 3 (Q3) counts above the negative probe geomean count (Supporting Information Figure 7A).

NanoString‐developed open‐source R packages GeoMxTools (v3.4.0) and NanoStringNCTools (v1.7.1; NanoString Technologies Inc) were used to analyse data from the NanoString GeoMx DSP instrument. GeoMx raw gene expression count files were loaded onto R, consisting of DCCs (expression count data and sequencing quality metadata), PKCs (probe assay metadata describing the gene targets present in the data), and annotation files (a spreadsheet containing sample metadata for each ROI); these files were combined as a single data object for subsequent steps of the GeoMx workflow. All ROIs were assessed for QC purposes to ensure sequencing quality as segment QC. Based on segment QC results, three ROIs were flagged and excluded from further analyses due to low stitched and alignment proportions [Slide 1, ROI 11; Slide 4, ROI 16; and Slide 4, ROI 27]. Standard probe QC steps were then applied to exclude outlier probes, and the limit of quantification (LOQ) was calculated to establish minimum quantifiable gene expression levels per segment. Quartile 3 (Q3) normalisation was applied as part of the guideline. Raw counts were exported and processed in Degust,^40^ a web‐based RNA‐seq analysis tool (v4.1.1) and for bulk analyses using the voom/limma pipelines.^41^, ^42^

### 2.6 Determination of normalisation method

NanoString guidelines recommend Q3 normalisation as a standard, however, there is also an option to normalise data using ROI nuclei count. Along with Q3 normalisation, normalisation to nuclei number was investigated via the NanoString R package.^43^ Several housekeeper genes (*RPLP0*, *RPS20*, *RPS29*, *SDHA*, and *YWHAZ*) were examined across all ROIs. Elevation of *YWHAZ*, *RPLP0*, *RPS20*, and *RPS29* was observed in all seminoma tumour ROIs, ‘Slide 4_06’ to ‘Slide 4_26’, however this appears to be a tissue‐specific effect, as the nuclei count for these ROIs falls from 241 to 387, close to or below the average nuclei count (Supporting Information Table 1 and Supporting Information Figure 7B). Q3 normalisation was more consistent across ROIs compared with nuclei count normalisation. Raw data were then uploaded into the Degust RNA‐Seq analysis tool,^40^ and resulting ‘counts per million’ via the voom/limma pipelines closely matched the Q3 normalisation values (Supporting Information Figure 7B), therefore this method was chosen for data analysis.

The processes of data analysis and resulting ROI annotations is delineated in the Results section based on established markers for specific cell types known to be present in the adult human testis and in TGCTs.

## 3 RESULTS

Spatial whole genome transcriptome analysis was performed on human testis tumour and tissue adjacent to the tumour from four patients (Table 1). An initial H&E histochemistry and immunostaining of non‐seminoma (to detect OCT4 and CD68) of independent sections from each tumour sample was employed to ascertain their overall cellular composition (Supporting Information Figure 2). Three patients had non‐seminoma tumours (NonSem1–3), and one patient sample contained only seminoma (Sem). Adjacent tissue was collected from testes containing tumour (from NonSem2‐3, Sem), deemed to be macroscopically normal, however, only NonSem2 adjacent tissue was histologically normal (Supporting Information Figure 4). NonSem3 adjacent tissue contained GCNIS and immune cell infiltrates (Supporting Information Figure 2), and Sem adjacent tissue had tubular atrophy and immune cell infiltrates (Supporting Information Figure 6). Spatial whole transcriptome RNA‐sequencing was performed on ROIs within tumour areas and adjacent tissue.

### 3.1 Region of interest annotation and validation

**FIGURE 2:**
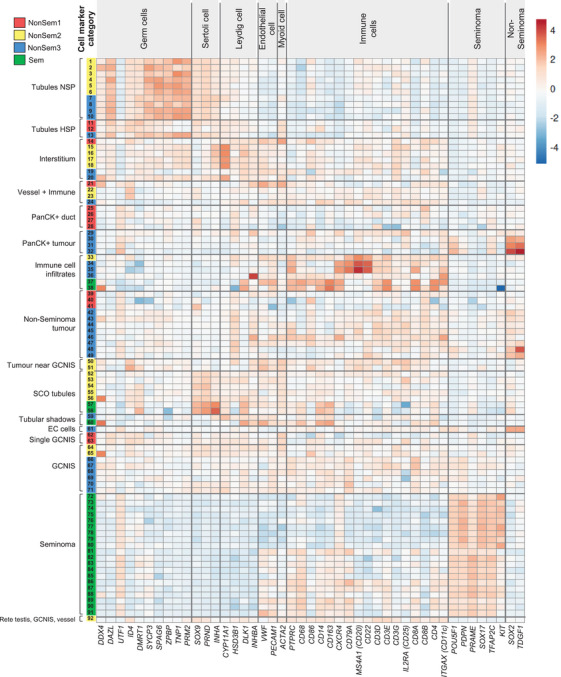
Validation of region of interest (ROI) annotations. Cell‐specific markers were used to confirm annotation of ROIs (far left labels). ROIs were listed in patient order (NonSem1, Red; NonSem2, Yellow; NonSem3, Blue; Sem, Green) within each annotation category. Cell marker categories (Top) reflect the grouped transcript markers listed at the bottom of the heatmap. Heatmap was made using ClustVis.^73^ Refer to Supporting Information Table 1 for additional information regarding ROIs.

At the time of NanoString analysis, the sections used to collect materials for RNA‐sequencing were stained with antibodies to detect CD45 and cytokeratin (Figure 1A). Immunofluorescence staining combined with H&E staining of the adjacent section was used to annotate and categorise ROIs (Figure 1A,B). ROIs were assigned to 16 categories based on histological assessment, including but not limited to regions of normal spermatogenesis (NSP), interstitium, PanCK+ ducts within non‐seminoma tumours, GCNIS and Sertoli cell only tubules (Figure 1C). ROIs were re‐labelled 1–92 based on category and patient ID (Supporting Information Table 1) and cell‐specific transcript markers were used to confirm their identity (Figure 2). Germline and somatic cell markers were selected using the Adult Human Testis Transcriptional Cell Atlas.^44^ Immune cell and TGCT tumour markers were identified from the literature.^15^, ^30^, ^33^ The expression profiles of cell‐specific markers validated the histological assessment of ROIs in this dataset. Germ cell‐related transcripts such as *DAZL* and *TNP1* were higher in normo‐spermatogenic (NSP) tubule ROIs (no. 1–10), along with levels of Sertoli cell markers *SOX9*, *PRND*, and *INHA*. The Leydig cell transcript *CYP11A1* was highly expressed in interstitium ROIs (no. 14–20), along with other Leydig, endothelial, myoid and immune cell markers, as expected. Seminoma‐containing ROIs (no. 72–91) had high levels of *POU5F1* (encodes OCT4), *PRAME*, *PDPN*, *SOX17*, and *TFAP2C*. Seminoma ROIs also exhibited immune cell transcripts, consistent with the presence of immune cell infiltrates often seen within tumour regions.^16^, ^30^, ^31^, ^32^, ^33^

To validate our protocols for assessing differences between ROIs, we compared NSP and interstitial regions in NonSem2 adjacent tissue (Supporting Information Figure 8A), as these have distinct and well‐characterised phenotypes. Amongst the 1167 differentially expressed genes (DEGs) identified (false discovery rate (FDR) < 0.05, LogFC1), 871 were downregulated in interstitial ROIs compared with NSP tubules, including *PRM2* and *TNP1* that are abundant in post‐meiotic germ cells. Amongst the 296 that were upregulated were Leydig cell transcripts, *CYP17A1* and *INSL3* (Supporting Information Figure 8B). DAVID functional annotation analysis provided further validation that pathways upregulated in the interstitium included steroid biosynthesis, lipid metabolism, cholesterol biosynthesis and innate immunity. Pathways upregulated in tubules with NSP included ‘spermatogenesis’, ‘cell differentiation’, ‘flagellum’ and ‘histones’ (Supporting Information Figure 8C).

### 3.2 Assessment of the GCNIS microenvironment

**FIGURE 3:**
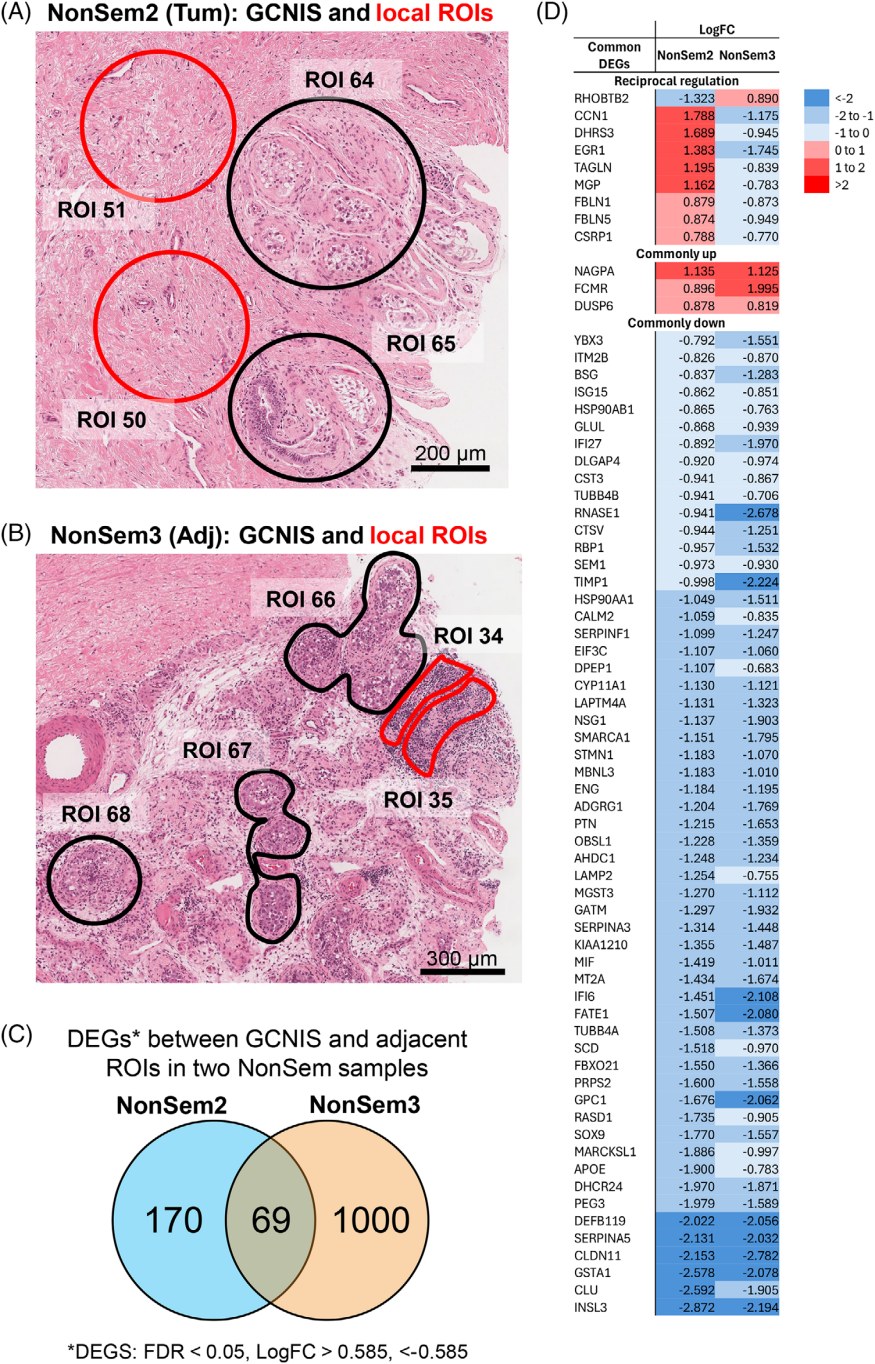
Analysis of regions of interest (ROIs) in close proximity to germ cell neoplasia in situ (GCNIS)‐containing ROIs. (A) Haematoxylin and eosin (H&E) images of HTCa2 tumour region representing ROI selection, with GCNIS‐containing ROIs (black circles, ROIs 64, 65) and adjacent tumour regions (red circles, ROIs 50, 51). Scale bar represents 200 µm. (B) H&E images of NonSem3 tissue adjacent to tumour region representing ROI selection, with GCNIS‐containing ROIs (black circles, ROIs 66–68) and adjacent immune cell infiltrates (red circles, ROIs 34, 35). Scale bar represents 300 µm. Comparison analysis within each patient comparing the adjacent ROIs with GCNIS ROIs was performed in Degust, with the output uploaded into Ingenuity Pathway Analysis (IPA). Cut‐offs within IPA were set to FDR < 0.05 and LogFC ← 0.585, >0.585 to identify DEGs. (C) Venn Diagram of number of DEGs identified in NonSem2 and NonSem3. (D) List of the common DEGs, with LogFC within patient subsets, with downregulated transcripts in blue, and upregulated transcripts in red. Venn diagram was created using JVenn.^74^

We examined the microenvironment within and surrounding GCNIS‐containing tubules as a strategy for discovering what factors might influence the fate and behaviour of these cells considered to be aberrant gonocytes that persist the adult testis. Histological assessment identified individual ROIs with tubules containing GCNIS in non‐seminoma samples: two in NonSem2 tumour (ROIs 64–65), and three in NonSem3 adjacent to tumour tissue (ROIs 66–68). *POU5F1* transcript (encoding OCT4) presence within these ROIs reinforced their classification as GCNIS‐containing ROIs (Figure 2). Next, ROIs in close proximity to each of these ROIs that were histologically similar to each other were identified. In NonSem2, two tumour ROIs (ROIs 50, 51) next to GCNIS were selected; these appeared fibrotic and contained scattered immune cells (Figure 3A). In NonSem3, two ROIs (ROIs 34, 35) containing immune cell infiltrates were selected (Figure 3B).

This allowed (1) comparison of transcripts between GCNIS‐containing regions and their histologically distinct adjacent regions, and (2) identification of common features and differences between the two biologically independent non‐seminoma tumours. Comparisons between the GCNIS and GCNIS‐adjacent ROIs within NonSem2 and NonSem3 were each made independently. Ingenuity Pathway Analysis (IPA) identified 239 and 1069 DEGs (adjacent ROIs vs. GCNIS) in NonSem2 and NonSem3, respectively (FDR < 0.05, LogFC > 0.585, ← 0.585; Figure 3C). In NonSem2, top differentially expressed genes within the GCNIS ROIs included the lipid metabolism gene *APOA1*, the steroid hormone synthesis enzyme‐encoding gene *CYP17A1*, and the cholesterol synthesis gene *DHCR24* (Table 3; full DEG list in Supporting Information Table 2). Further, the top pathways identified between GCNIS and GCNIS‐adjacent ROIs with a negative *z*‐score (−log(*p*‐value), *z*‐score < (−2)) included cholesterol biosynthesis pathways, indicating a higher level of cholesterol biosynthesis‐related transcripts within the GCNIS‐containing ROIs (Supporting Information Table 3). In NonSem3, the top DEGs in immune cell infiltrates were *MS4A1* and *CD22*, both B cell markers (Supporting Information Table 4), and pathways included ‘Neutrophil Degranulation’ and ‘Natural Killer Cell signalling’ (Supporting Information Table 5). Interestingly, ‘IL12 signalling and production in macrophages’ was common within the top 15 pathways in both patient comparisons.

**TABLE 3:** Top 10 regulated genes in NonSem2 germ cell neoplasia in situ (GCNIS) and tumour near to GCNIS.

Gene ID	FDR	LogFC	Avg CPM GCNIS ROIs	Avg CPM tumour ROIs
**Top 10 upregulated DEGs in GCNIS‐containing regions of interest (ROIs)**				
PTGDS	1.41E−12	−3.861	1369.62	107.04
CLU	4.63E−09	−2.592	1092.97	178.08
CYP17A1	4.63E−09	−2.806	321.95	47.10
CLDN11	6.73E−08	−2.153	389.74	94.10
GSTA1	7.62E−08	−2.578	364.05	60.93
MZT2B	1.73823E−06	−1.942	28531.12	7533.14
DHCR24	2.03055E−06	−1.970	217.48	57.42
MARCKSL1	2.78853E−06	−1.886	213.88	60.93
C4B	4.62634E−06	−2.096	187.25	46.00
APOA1	1.07938E−05	−2.495	141.43	26.39
**Top 10 upregulated DEGs in tumour next to GCNIS**				
LUM	1.76E−09	2.213	150.06	738.97
ADH1B	2.61E−09	2.255	65.38	334.52
COL3A1	7.62E−08	1.879	163.14	631.63
TAAR9	1.44E−07	2.106	28.79	129.84
CCN1	1.74E−07	1.788	81.69	299.74
NR2F1	2.34E−07	1.916	59.26	237.65
IGKC	3.74E−07	3.080	1226.30	8073.67
DHRS3	8.57E−07	1.689	64.54	220.62
C7	2.67688E‐06	1.552	308.05	963.34
CLDN5	5.33936E−06	1.733	33.59	119.53

To identify common features in the GCNIS microenvironments of different patients, DEGs were obtained by comparing the GCNIS and GCNIS‐adjacent ROIs from each NonSem2 and NonSem3. There were 69 DEGs that were commonly identified in both (Figure 3C). Three DEGs were commonly higher in both GCNIS‐adjacent ROIs relative to the GCNIS‐containing ROIs, 57 were commonly lower, and nine DEGs exhibited reciprocal regulation in the comparison between NonSem2 and NonSem3 (Figure 3D).

Upstream regulator analysis identified the components of the TGFβ family within NonSem2 (TGFBR2, SMAD4; prediction activated, *z*‐score >2; Supporting Information Table 6) and NonSem3 (TGFβ family, TGFβ1, SMAD3; prediction inhibited, *z*‐score ← 2; Supporting Information Table 7). It is striking that within the list of 69 common DEGs, *SERPINA5*, *CLDN11*, *INSL3*, and *CYP11A1* have been identified as activin A‐target genes in transgenic mouse models of altered activin A and in ex vivo cultures of foetal mouse gonads.^45^, ^46^ These were all higher in the CGNIS ROIs, indicating that activin stimulation may be important to maintaining the environment for these genes which are produced in somatic cells of the testis.

### 3.3 Patient‐specific immune cell profiles in non‐seminoma tumours

**FIGURE 4:**
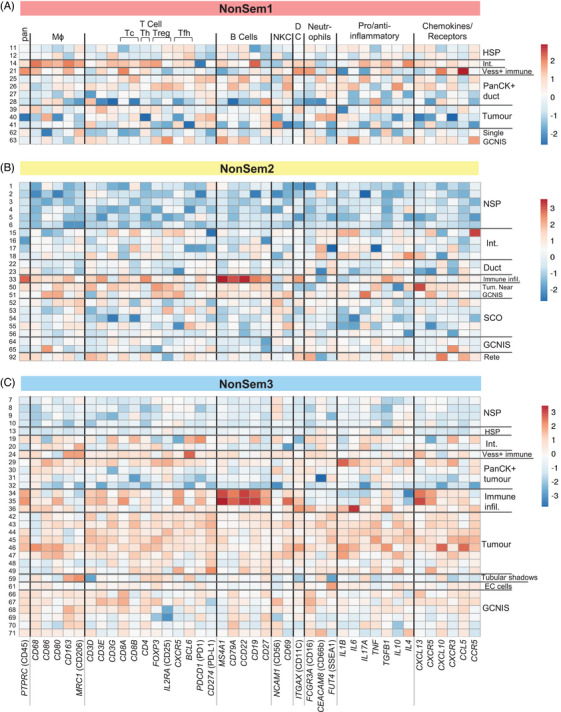
Heatmap of immune cell markers in non‐seminoma patients. Relative expression of immune cell markers are displayed via heatmap in individual regions of interest (ROIs) from each non‐seminoma patient sample: (A) NonSem1, (B) NonSem2 and (C) NonSem3. Individual rows represent an ROI, numbers on the left match those in Figure 2 heatmap, and the corresponding annotations are listed on the right. Immune cell subtype groupings are indicated at the top, with the corresponding gene name at the bottom of the columns. Immune subset abbreviations are as follows: DC, dendritic cell; mϕ, macrophage; NKC, natural killer cell; pan, pan‐immune cell marker; Tc, cytotoxic T cell; Tfh, follicular T helper cell; Th, T helper cell; Treg, regulatory T cell;. ROI annotation abbreviations are as follows: EC, embryonal carcinoma; HSP, hypospermatogenesis; infil, infiltrates; Int, interstitium; NSP, normo‐spermatogenesis tubules; Rete, rete testis; SCO, Sertoli cell only tubule/s; Tum, tumour; Vess, vessel. Heatmaps were generated using ClustVis.^73^

The immune cell profiles within and between tumours from individual patients contributes to TGCT heterogeneity,^16^, ^30^, ^31^, ^32^, ^33^ therefore we mapped transcripts encoding several immune cell markers: macrophage, T cell, B cell, natural killer cell (NKC), DCs and neutrophil subsets (Figure 4). Amongst all non‐seminoma samples, the immune cell transcript signal was lowest in ROIs with NSP. Immune cell profiles within NonSem1 ROIs were heterogeneous, with the strongest signal for immune cell markers in the ROI containing blood vessels (Figure 4A). In NonSem2, tissue adjacent to the tumour was histologically normal (ROIs 1–6 with NSP; ROIs 15–18 interstitium (Int); Supporting Information Figure 4), and immune cell profiles reflected this, with little to no immune cell markers in NSP ROIs, and moderate expression in interstitial ROIs (Figure 4B). The immune cell‐containing infiltrate ROIs (nos. 33, 34 and 35) in both NonSem2 and NonSem3 (Figure 4B,C) had high expression of B cell markers (*MS4A1*, encoding *CD20*; *CD79A*; *CD22*; *CD19*). In NonSem3, immune cell regions (ROIs 34–36) also distinctively high levels of the transcript encoding the B cell attractant chemokine CXCL13 and its receptor, CXCR5 (Figure 4C). NonSem3 tumour regions (ROIs 42–49) demonstrated broad expression of most immune cell subtype markers, with higher levels of T cell compared with B cell markers.

### 3.4 Immune cell profiles in tissue adjacent to seminoma

**FIGURE 5:**
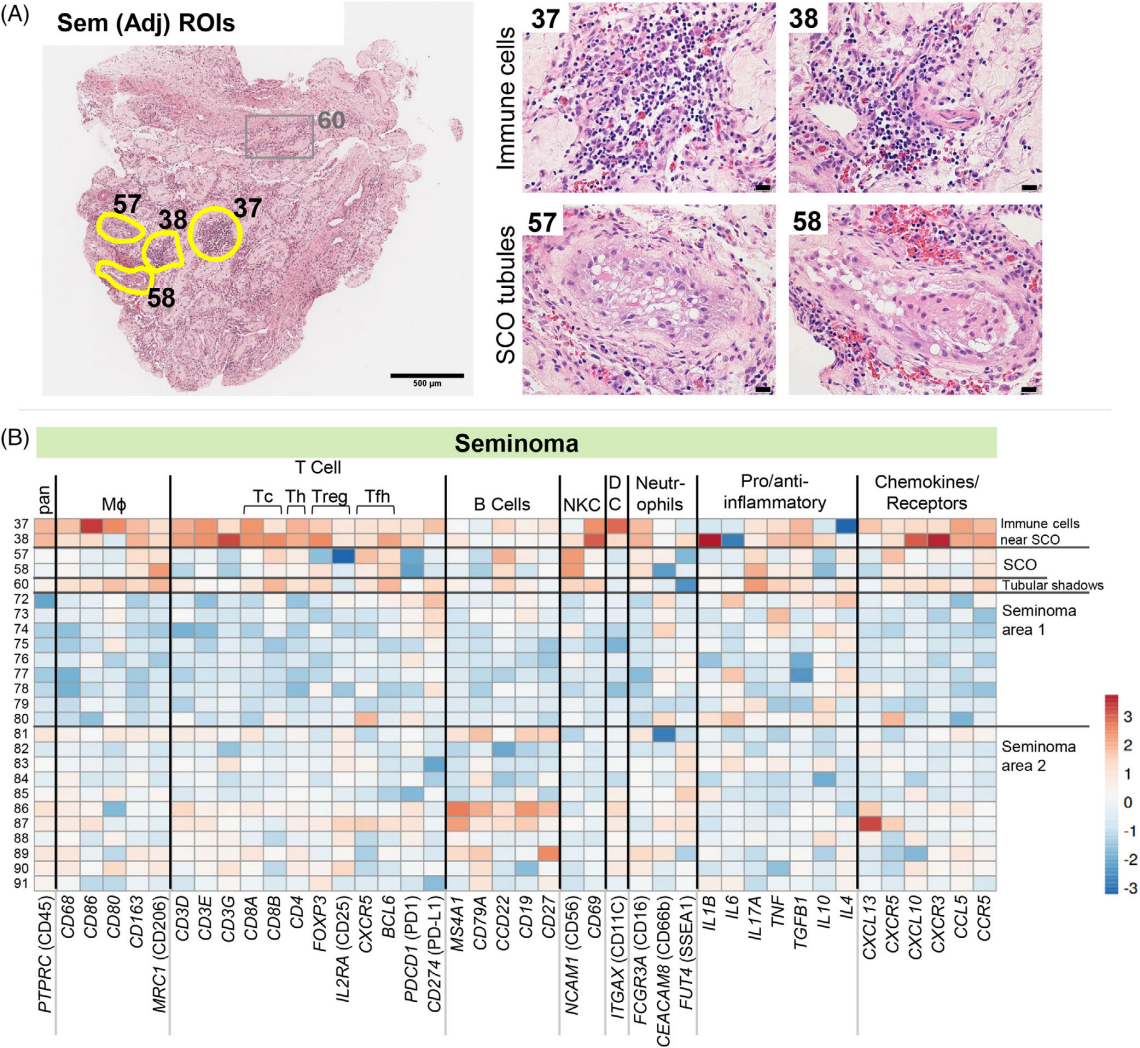
Immune cell profiles in tissue adjacent to seminoma tumour. (A) Haematoxylin and eosin (H&E) staining of Sem adjacent tissue. Approximate outlines of regions of interest (ROIs) indicated on left hand (low mag) image (scale bar, 500 um). High magnification images of yellow‐outlined ROIs are presented on the right, with immune cell infiltrate ROIs on top panel, and Sertoli cell only (SCO) tubules on bottom panel (scale bars 20 um). (B) Heatmap of immune cell subtype markers across all Sem ROIs.

In tissue adjacent to the seminoma tumour (Supporting Information Figure 5A), amongst the five ROIs selected for spatial transcriptomic analysis, two harboured immune cell infiltrates located next to two SCO tubules, and a region of tubular shadows (ROIs 37, 38, 57, 58, and 60; Figure 5A). There was no histological evidence of seminoma or GCNIS cells within this tissue section (Supporting Information Figure 6A), an observation reinforced by the absence of germ cell markers within the SCO ROIs (Figure 2). Immune cell infiltrates near the SCO tubules had higher levels of macrophage, T cell, NKC and DC markers and several chemokine and receptor transcripts, compared with the low abundance of B cell markers (Figure 5B). Interestingly, ROI 38 (immune cell infiltrate near SCO), had high levels of transcripts encoding the pro‐inflammatory IL1B, the T cell and NK cell chemoattractant CXCL10, and its receptor CXCR3. Immune cell transcripts were present within SCO tubule ROIs, albeit at lower levels compared with the immune cell infiltrate ROIs.

### 3.5 Heterogeneity in seminoma tissue from an individual patient

**FIGURE 6:**
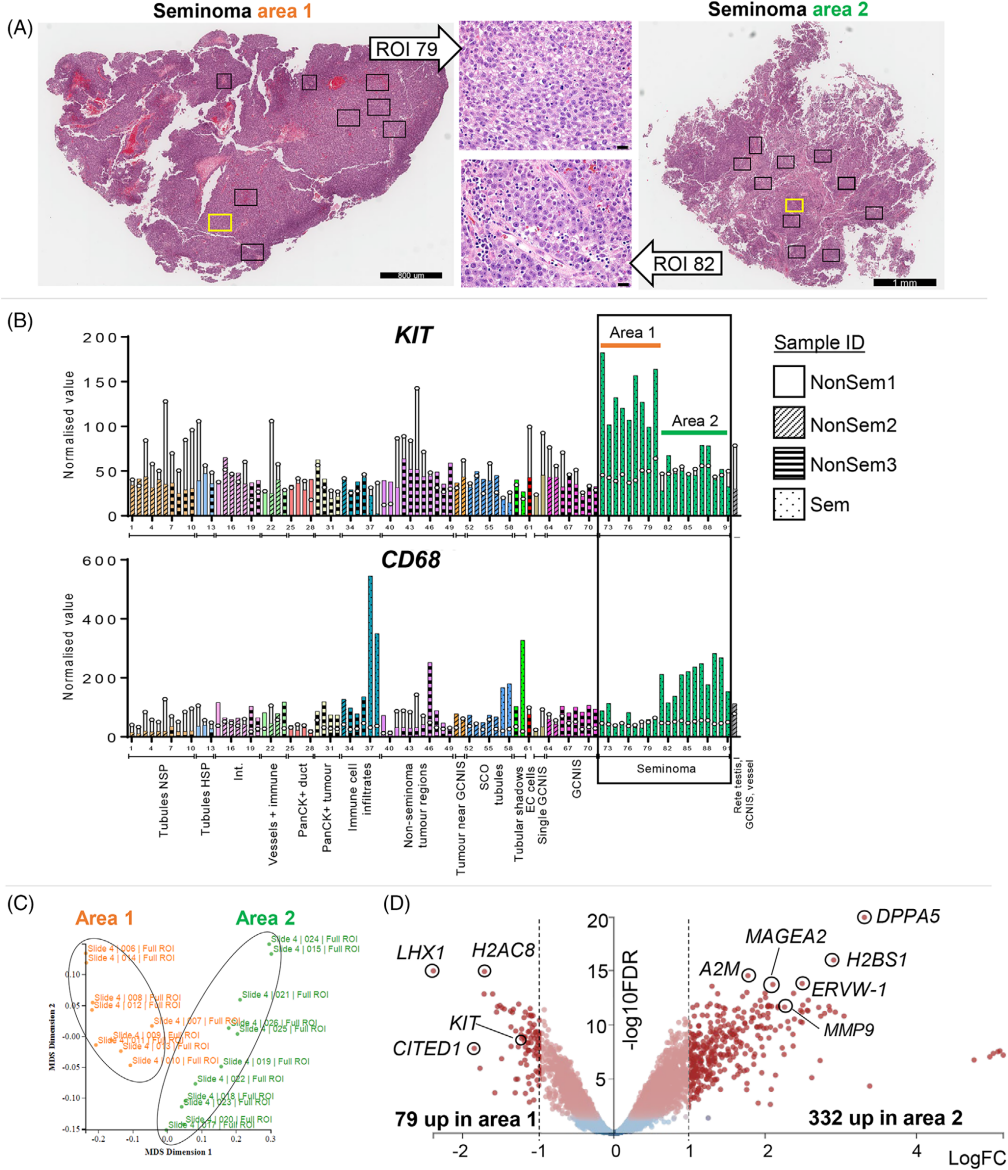
Seminoma areas from single patient are transcriptionally different. (A) Haematoxylin and eosin stain of adjacent serial section to NanoString experimental sections from area 1 and area 2 seminoma tumour. Boxes indicate approximate regions of interest (ROIs) selected for spatial whole transcriptomic analysis. High magnification images (scale bar 20 µm) in the middle panel correspond to yellow ROI boxes. (B) Normalised values of *KIT* in individual ROIs (1–92 of whole dataset). Colours represent annotation groupings, and bar markings represent sample ID. Green bars represent Sem, with the black box outlining seminoma tumour ROIs (area 1, orange line; area 2, green line). Open bars and circles represent the limit of quantification, where a value above is considered a robust signal. (C) MDS plot generated within Degust software of Area 1 (orange) and Area 2 (green) ROIs. (D) Volcano plot demonstrating transcripts differentially expressed by FDR < 0.05 (red dots), and greater than LogFC(1), or less than LogFC(‐1) (dashed lines).

The initial histological assessment of the seminoma sample indicated there were two distinct areas, and this was reinforced when spatial transcriptomics identified significant differences in cell‐specific markers (Figure 2). Nine and 11 ROIs were selected and analysed in each of these two distinct areas (Figure 6A). As predicted from histological observations, transcripts encoding immune cell markers such as *CD68*, were generally higher in area 2 (ROIs 72–80; Figures 5B and 6B), while the early germ cell and seminoma marker, *KIT*, was higher in section 1 (ROIs 81–91, Figure 6B). Despite the prevalence of seminoma cells in both regions, the two areas were transcriptionally distinct, as evident by the multidimensional scaling (MDS) plot groupings of ROIs (Figure 6C). B cell transcripts were higher in a subset of the area 2 ROIs, compared with markers of other immune cell types.

A comparison between the two seminoma areas identified 411 DEGs (FDR < 0.05, LogFC > 1, ← 1), with 332 higher and 72 lower in area 2 (with immune cells) versus area 1 (Figure 6D and Supporting Information Table 8). Transcripts higher in area 1 encode transcription factors *LHX1* and *CITED1*, the tyrosine kinase receptor *KIT*, and the histone component *H2AC8*. Elevated transcript levels in area 2 included of pluripotency‐associated *DPPA5*, testis cancer antigen *MAGEA2*, matrix metalloproteinase *MMP9*, and histone component *H2BS1*. These findings provide evidence of fundamental differences in seminoma cells in areas with and without immune cell infiltrates within the same tumour. Unsurprisingly, the top pathways identified from DEG analysis using IPA were most commonly related to immune cell signalling and biology (Supporting Information Table 9), while top‐ranked upstream regulators (activated, *z*‐score >2) included cytokines interferon gamma (IFNG), tumour necrosis factor (TNF) and interleukin 4 (IL4), and TGFβ1 (Supporting Information Table 10).

### 3.6 TGFβ signalling pathway machinery in seminoma tumour areas

**FIGURE 7:**
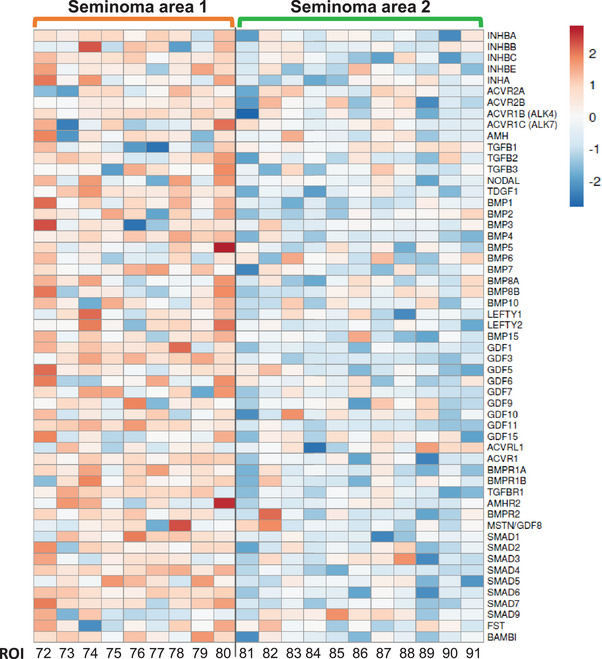
TGFβ superfamily member profiles in seminoma regions of interest (ROIs). A heatmap of transcript levels of TGFβ superfamily ligands, receptors, intracellular machinery and selected inhibitors in the seminoma area 1 (orange) and area 2 (green) tumour ROIs.

The presence and potential involvement of TGFβ superfamily members in TGCT aetiology has been demonstrated.^38^, ^47^, ^48^, ^49^, ^50^ *KIT* and *MMP9* transcripts are modulated by activin A, a ligand of the TGFβ superfamily, in mouse and human models.^45^, ^50^, ^51^, ^52^ Because these transcripts were identified as differentially expressed between area 1 and area 2 of the seminoma tumour ROIs (Figure 6D), and TGFβ1 was identified as an upstream regulator by IPA, we next profiled TGFβ superfamily machinery within the seminoma area 1 and area 2 ROIs. The relative abundance of TGFβ superfamily machinery in area 1 compared with area 2 included transcripts encoding ligands, receptors, intracellular SMADS, and some pathway inhibitors (Figure 7). Within area 1, there was notable heterogeneity between individual ROIs, corresponding to different levels of ligand and receptor transcripts (e.g., BMP5, AMHR2 are particularly high in ROI 80).

## 4 DISCUSSION

This study demonstrates the potential for using spatial transcriptomics to provide information about nature of tumours within individual patient samples that cannot be discerned by conventional histological analysis. TGCT heterogeneity is particularly challenging when examining non‐seminoma tumours in particular, because they often contain multiple differentiated cell types, however as demonstrated here, seminomas are also heterogeneous despite being labelled ‘pure’ by the pathologist. The combined evaluation of transcriptomes and morphology in discrete TGCT tissue regions can identify potential signalling mechanisms and the potential role of immune cells within such heterogeneous tumours.

We still do not understand how GCNIS precursors first emerge, despite evidence of combined genetic and environmental cues. Their developmental trajectory into different tumour subtypes is even more opaque, due to the prolonged timeline during which this is likely to occur. Candidate signalling pathways that have been implicated include WNT, FGF and the TGFβ superfamily,^29^, ^47^, ^48^, ^49^, ^53^ while the potential for immune cells to influence the fate of these gonocyte‐like that are present at the cord basement membrane has attracted significant interest.^16^, ^54^, ^55^ Evidence implicating each of these, or other processes, could eventually lead to strategies to minimise the conversion of these cells into tumours.

In the present study we sought to learn about factors in the local environment that could be influencing GCNIS fate by evaluating transcripts in GCNIS‐containing ROIs and in their neighbouring ROIs (Figure 3). Our insights are limited due to the small sample number, comprised two GCNIS tubules and adjacent ROIs from two independent non‐seminoma samples. However, in both patient samples the microenvironment adjacent to the GCNIS tubules contained higher levels of cholesterol and steroid biosynthesis‐related transcripts, an outcome intriguingly consistent with the previous identification of lipid droplets within tubules containing GCNIS.^56^ As expected, the majority of transcripts present at lower levels in GCNIS‐adjacent ROIs are Sertoli and Leydig cell products (e.g., *SOX9*, *SERPINA5*, *INSL3*, *CLU*). The three transcripts that were higher in adjacent ROIs, *NAGPA*, *FCMR*, *DUSP6*, are associated with different aspects of protein processing. *NAGPA* encodes N‐acetylglucosamine‐1‐phosphodiester alpha‐N‐acetylglucosaminidase, an enzyme that selectively removes the GlcNAc carbohydrate and thereby contributes to protein delivery to lysosomes. *FCMR* codes for the high‐affinity Fc receptor for both membrane‐bound and secreted immunoglobulin M (IgM) and has been implicated in regulating both T and B cell functions, modulation of myeloid responses in vivo and in vitro, as well as facilitating IgM–antigen complex degradation by directing their movement into endosomes.^57^ *DUSP6* encodes a dual specificity protein phosphatase subfamily member that has wide‐ranging effects on cell behaviour by acting on extracellular signal‐regulated kinases (ERK) proteins in the mitogen‐activated protein (MAP) kinase superfamily; mutations in *DUSP* have been linked to hypogonadotropic hypogonadism.^58^ For each of these three genes, the functional implications of their relatively high expression in sites adjacent to GCNIS are currently unknown, although we speculate that they could influence immune cell functions and/or antigen presentation. Identifying activin A‐target genes amongst the DEGs common to both, along with components of the TGFβ family as upstream regulators, provides intriguing evidence that this pathway is central to the processes that sustain gonocyte‐like germ cells in a maturing testis environment.

The finding that TGFβ signalling machinery and activin downstream target gene expression was much lower in the region of seminoma that contained immune cell infiltrates (Sem area 2) gives a strong indication that immune cells may influence the fate of these neoplastic germ cells. The principle that BMP signalling downregulation is a pre‐requisite for seminoma transformation into non‐seminoma cell type was demonstrated using transplanted TCam‐2 seminoma cells by Nettersheim et al.^38^ It is therefore logical to consider whether immune cell infiltrates, such as the subtypes identified by the presence of B cell‐related transcripts, create a microenvironment that enables seminoma cells to transform into a more malignant tumour subtype.

The documented complexity and variety of immune cell subtypes within seminomas and non‐seminomatous TGCTs has been observed predominantly using immunohistochemistry methods.^30^, ^32^, ^33^, ^59^ The presence of multiple immune cell subtype transcripts in ROIs of the three non‐seminoma patient tumours investigated here offers an unbiased perspective, as it is not limited by antibody availability or fixation approaches. It was interesting that our data indicate higher levels of B cell‐expressed transcripts compared with those typical of T cells in non‐seminoma tumour immune cell infiltrate ROIs. Most frequently, macrophages and T cells are reported as the dominant immune cell populations in non‐seminoma tumours, with B cells detectable and highest in mixed TGCT compared with seminoma and embryonal carcinoma.^30^, ^59^ Transcripts encoding factors relating to B cell differentiation and activation were abundant in both samples within the ROIs adjacent to GCNIS in NonSem2 and NonSem3, suggesting a functional interaction exists between the GCNIS and B cells. Based on the results presented here, we expect that knowledge from unbiased spatial analyses of immune cells in additional patient samples will reveal their impact on GCNIS fate.

Examining the tissue adjacent to tumour regions in TGCT samples may identify processes influencing their formation and maintenance. This methodology also enables us to develop a more refined understanding of seminomas which appear histologically homogeneous and have largely been treated as such. For example, enabling identification of additional markers or risk factors relating to differences between two seminoma subtypes, such as those identified in the large computational study of 64 seminoma samples using The Cancer Genome Atlas. The first harbours a higher pluripotency status with a bigger neutrophil population, and the second with higher *FOXA2* and a ‘reprogrammable’ phenotype.^55^, ^60^, ^61^ We did not identify ROIs with significant levels of *FOXA2*. The seminoma tumour in our dataset appears closer to the first subtype, however the presence of immune cells yields further differences in transcript signatures between the two areas within this individual patient.

Additional approaches have identified regional heterogeneity within seminomas. One study used laser microdissection followed by mRNA profiling using nCounter‐based gene expression profiling to identify transcriptional differences between non‐metastatic and metastatic seminomas, comparing the centre of the tumour and the tumour front of these two tumour stages.^62^ Most differential gene expression was documented in comparisons between seminoma tumour stages, with both exhibiting a tumour front that had a distinct gene profile relative to the central regions. In metastatic tumour front tissue, transcripts relating to immune cell pathways, metabolism, inflammation and angiogenesis, and to the IL6 signalling pathway were higher when compared with non‐metastatic seminoma tumour fronts. In another study employing scRNA‐Seq, a single patient seminoma sample was shown to contain with two transcriptionally distinct tumour subtypes; one expressed the WNT signalling transcription factor *TCF7L1* (encodes T cell factor 7 like 1; WNT signalling mediator), and the second expressed *SCG3* and *SV2C*.^63^ This increasing evidence of seminoma tumour heterogeneity between patients, and within a patient, including in our dataset, highlight the need to establish a solid understanding of what features of heterogeneity are functionally important for clinical management.

A striking difference between area 1 and area 2 of the seminoma tumour specimen was the higher levels of transcription factor *LHX1* and the tyrosine kinase receptor *KIT* in area 1 ROIs. LHX1 function in seminoma has not been studied, however, it is a marker of the earliest undifferentiated spermatogonial stem cells in the developing mouse testis.^64^ Further, *LHX1* expression correlated with poor overall survival in uterine corpus endometrial carcinoma cells and tissue within the Cancer Genome Atlas.^65^ KIT production in primordial germ cells is required for their survival and migration to the genital ridge during embryogenesis.^66^ The expression of KIT is a hallmark of seminomatous TGCT, reflecting their early germ cell origin, and its levels can be modulated by exogenous activin A in both seminoma‐derived TCam‐2 cells, with an increase in transcript levels,^52^, ^67^ and in seminoma tumour fragment hanging drop cultures, with decreased transcript and protein levels.^50^

Neutrophil degranulation and Interferon gamma signalling were each identified as top‐regulated pathways in area 2 of the seminoma sample along with higher levels of immune cell subtype markers, indicating a function for localised, enhanced neutrophil activity. Neutrophils are abundant, first responder cells in the innate immune response to insults and pathogens, however, they may be reprogrammed into pro‐tumourigenic neutrophils.^68^, ^69^ Increased levels of neutrophils in non‐seminoma tumours have been associated with metastasis and poor survival outcomes in patients.^70^ Activated neutrophils can release cytokines, inflammatory mediators, and stimulate T cells. They also undergo degranulation, a complex process which releases proteins such as the matrix metalloproteinase MMP9, which is itself associated with tumour progression and angiogenesis.^71^, ^72^ *MMP9* is expressed in seminomas, and exposure of the TCam‐2 cell line to activin A results in increased *MMP9* transcript levels.^51^ The higher levels of neutrophil markers and *MMP9* in area 2 of the seminoma sample compared with area 1 in the present study highlights the presence of two distinct microenvironments. Our previous work showed that, along with pro‐inflammatory (IL‐1β, IL‐6, TNF‐α) and Th1‐related cytokines (IL‐2, IFN‐γ), transcripts encoding Treg‐related cytokines TGF‐β and IL‐10 were significantly higher in TGCT compared with testis tissue regions with NSP or non‐neoplastic inflammation. In addition to the identification of Tfh cells and B cell clusters in seminoma samples, increased transcript levels of supporting chemokines have been identified in testicular germ cell neoplasia, that is, CXCL‐13 and CCL‐5.^16^, ^30^, ^32^ This further underlines the tremendous differences in the microenvironments of seminomas compared with NSP, non‐neoplastic testicular inflammation, and non‐seminoma tumours.

In conclusion, this study demonstrates the power of employing spatial transcriptomics to study different regions of a tumour, including as a tool to explore the diversity of microenvironment and immune cell populations in TGCTs. Although this technology is limited to providing a snapshot of the tumour at time of excision, we have demonstrated approaches that will enable its application to understand tumour aetiology as further samples are explored by us and others. While the technology employed in this study lacks resolution at the single cell level, we were able to combine expert histological analysis with localised transcriptome mapping to search for clues about the factors which sustain and transform GCNIS cells. Ultimately the improved resolution and comparison with a greater number of samples has the potential to yield personalised approaches to therapy and to inform strategies for ongoing management of patients with TGCTs.

## AUTHOR CONTRIBUTIONS

**Sarah C. Moody**: Design and implement experiments; data analysis and manuscript writing. **Daniela Fietz**: Design experiments; data analysis and manuscript review. **Benedict Nathaniel**: Data analysis and manuscript review. **Mark Frydenberg**: Sample provision and manuscript review. **Ben Tran**: Sample provision and manuscript review. **Hans‐Christian Schuppe**: Design experiments; data analysis and manuscript review. **Kate L. Loveland**: Design experiments; data analysis and manuscript writing.

## CONFLICT OF INTEREST STATEMENT

Sarah C. Moody, Daniela Fietz, Benedict Nathaniel, Mark Frydenberg, Hans‐Christian Schuppe and Kate L. Loveland declare no conflicts of interest.

Ben Tran receives research funding from Amgen, Astellas, AstraZeneca, Bayer, BMS, Genentech, Ipsen, Janssen, Pfizer, Movember and MSD. Ben Tran is an Honorarium of Amgen, Astellas, AstraZeneca, Bayer, BMS, Ipsen, Janssen, Merck, MSD, Pfizer, Sanofi, Tolmar. Ben Tran has consulting/advisory positions with Amgen, Astellas, AstraZeneca, Bayer, BMS, Ipsen, IQVIA, Janssen, Merck, MSD, Novartis, Pfizer, Roche, Sanofi and Tolmar.

## Supporting information



Supporting Information

Supporting Information

Supporting Information

Supporting Information

Supporting Information

Supporting Information

Supporting Information

Supporting Information

Supporting Information

Supporting Information

Supporting Information Figure 1

Supporting Information Figure 2

Supporting Information Figure 3

Supporting Information Figure 4

Supporting Information Figure 5

Supporting Information Figure 6

Supporting Information Figure 7

Supporting Information Figure 8
