# Supplementary figures and images for: Spatial transcriptomics mapping of immune cell and TGFβ signalling pathway heterogeneity in testicular germ cell tumours
Source: Andrology. 2025 Jul 22;14(1):210–27. doi: 10.1111/andr.70100 (PMC12670472; doi:10.1111/andr.70100)

## Section 1

NonSem1 A

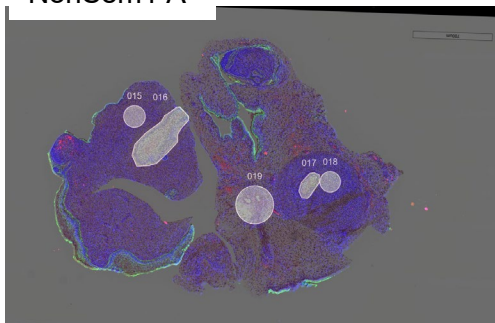

## Section 2

NonSem1 B

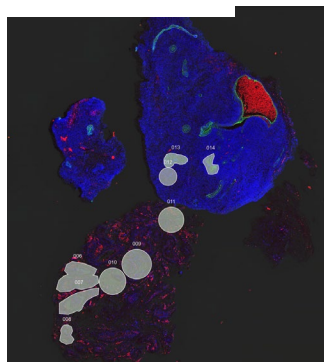

## Section 3

NonSem 2 Adj A

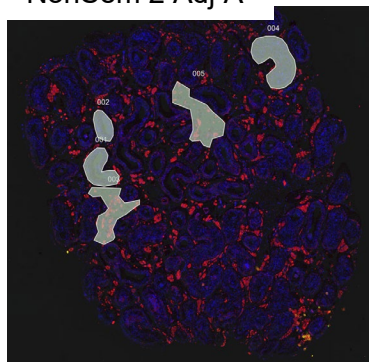

NonSem 2 Adj B

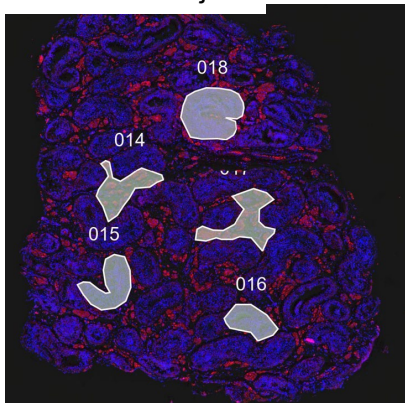

Supplement: Supplementary file 11 — Supporting Information Figure 1 [file ANDR-14-210-s003.pdf]

**NonSem3, Adj A**

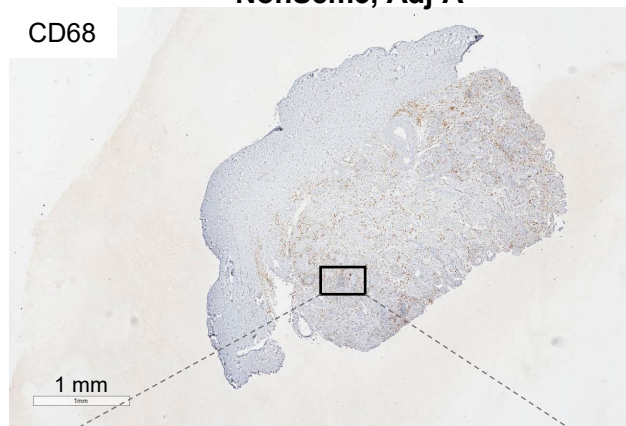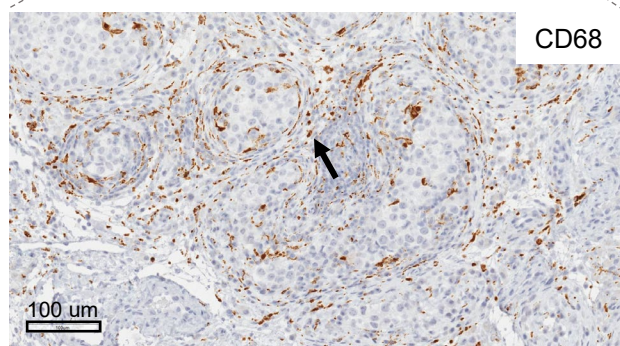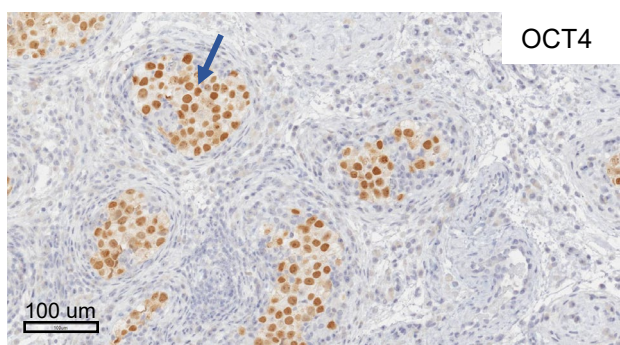

**NonSem3, Adj B**

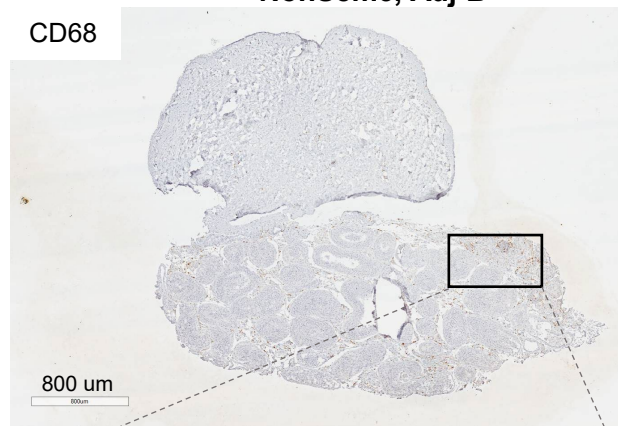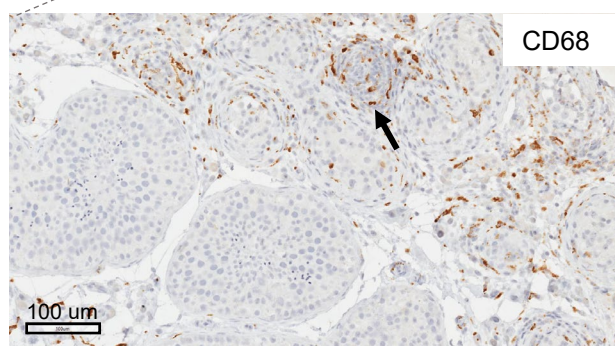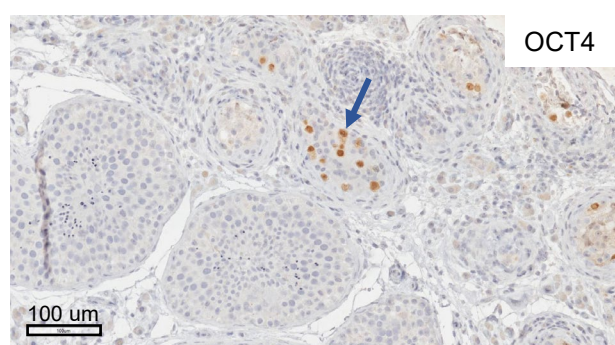

Supplement: Supplementary file 12 — Supporting Information Figure 2 [file ANDR-14-210-s002.pdf]

NonSem1 Tum A

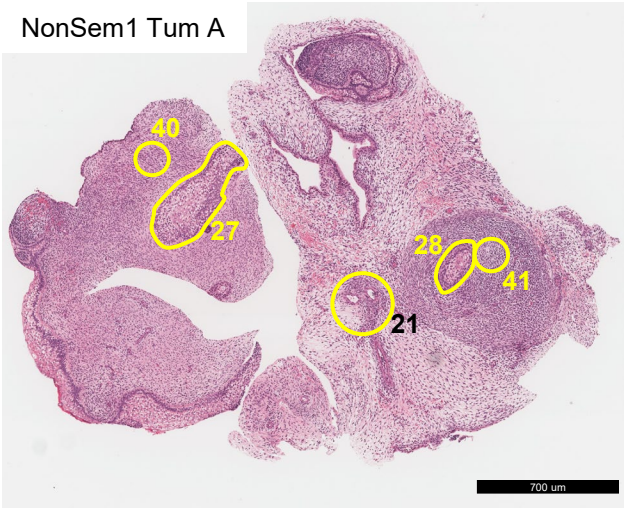

NonSem1 Tum B

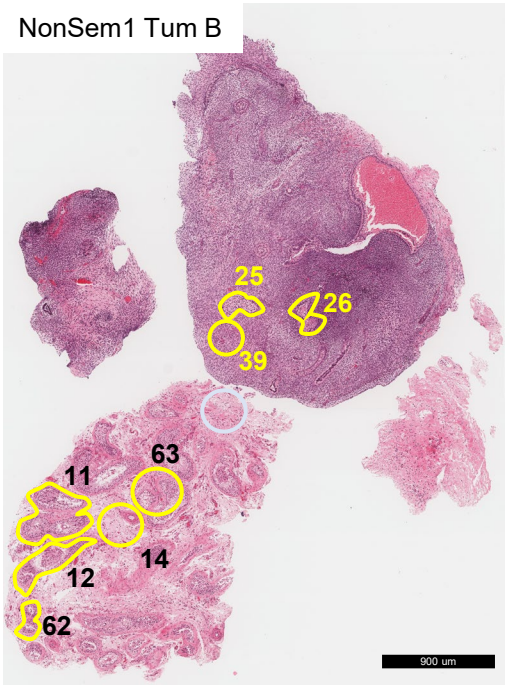

Supplement: Supplementary file 13 — Supporting Information Figure 3 [file ANDR-14-210-s009.pdf]

NonSem2 Adj A

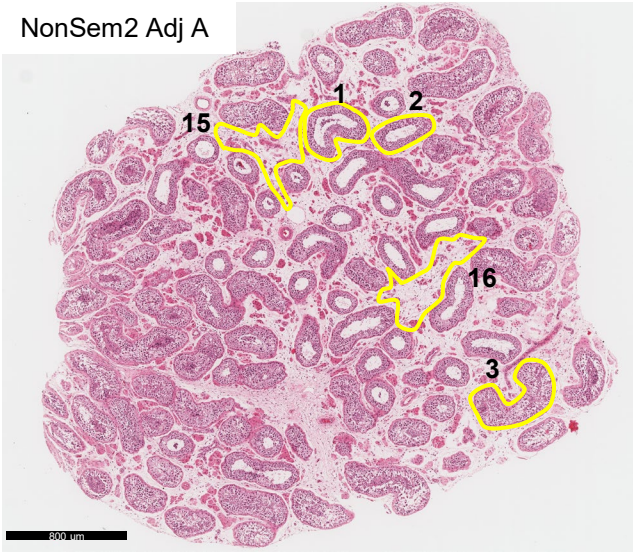

NonSem2 Adj B

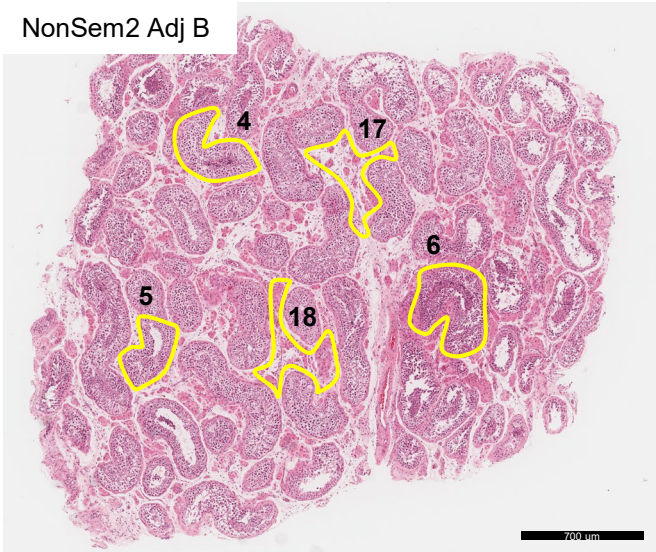

NonSem2 Tumour

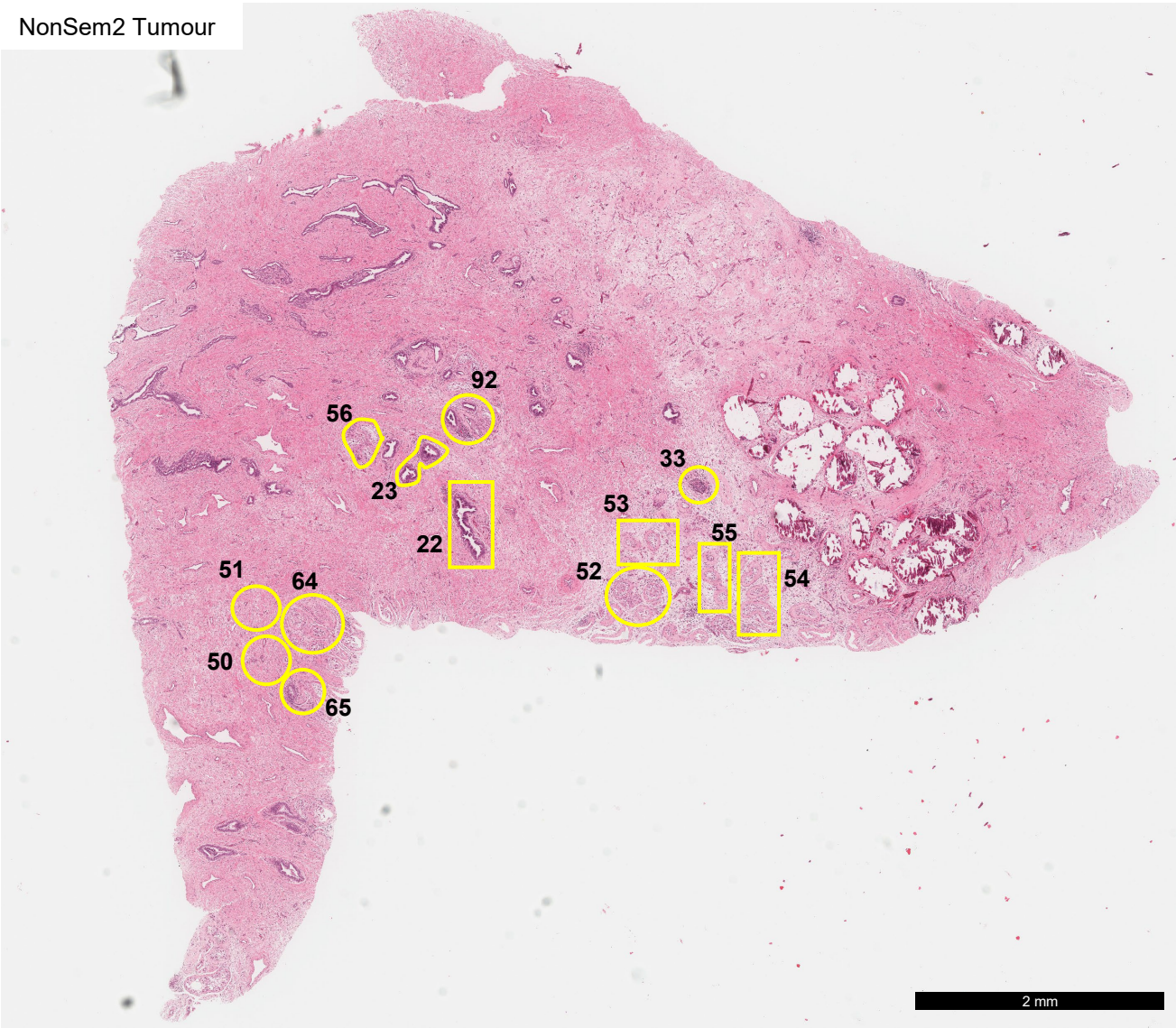

Supplement: Supplementary file 14 — Supporting Information Figure 4 [file ANDR-14-210-s006.pdf]

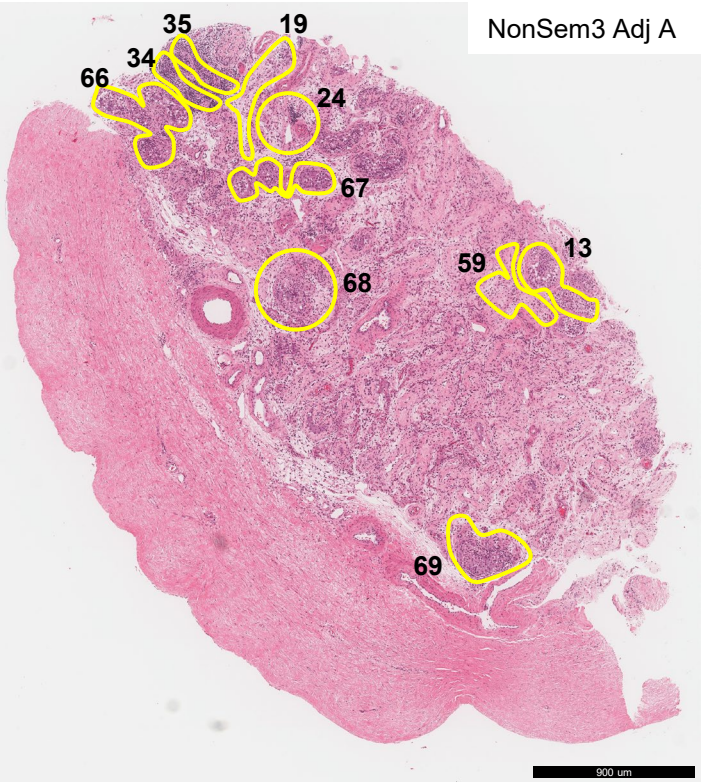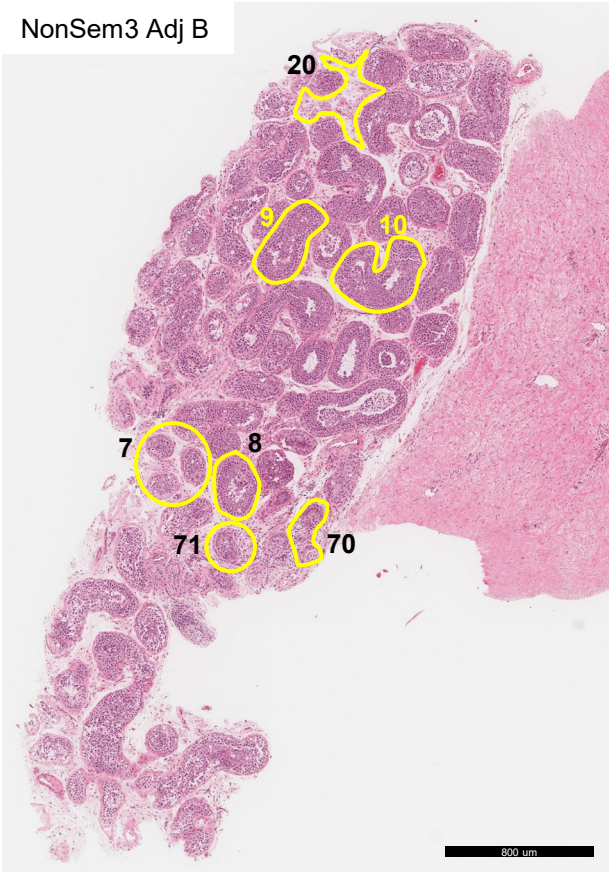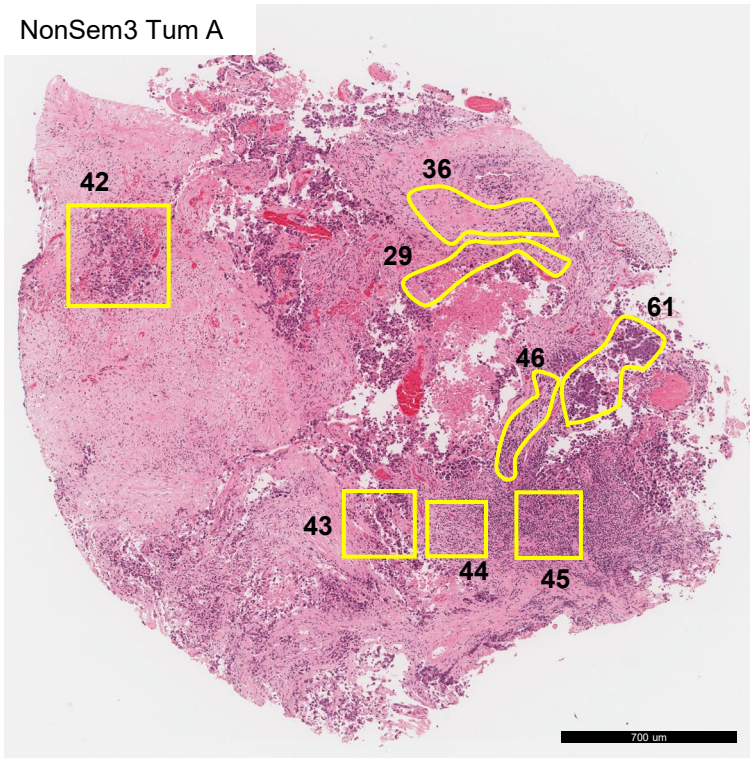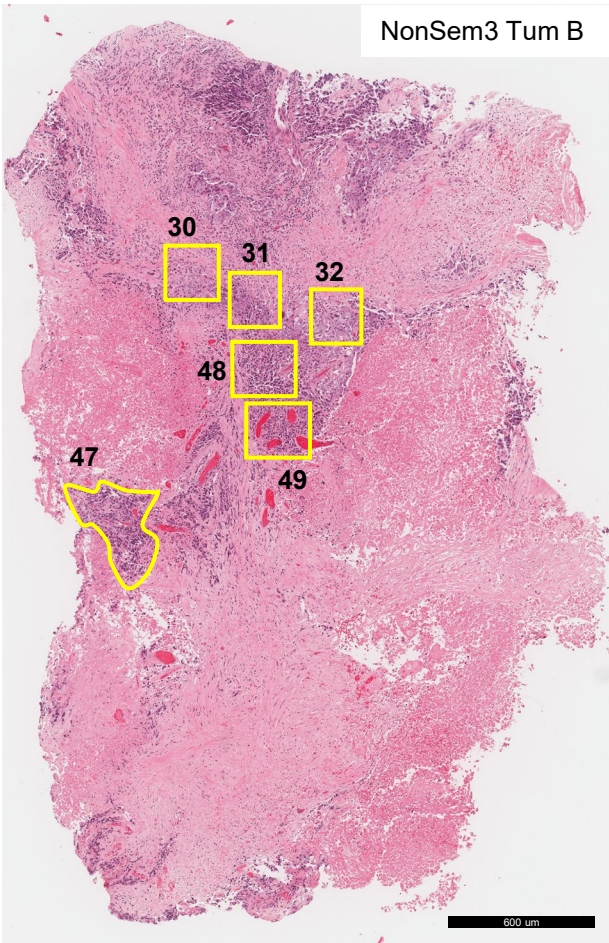

Supplement: Supplementary file 15 — Supporting Information Figure 5 [file ANDR-14-210-s013.pdf]

**A**

Sem Adj

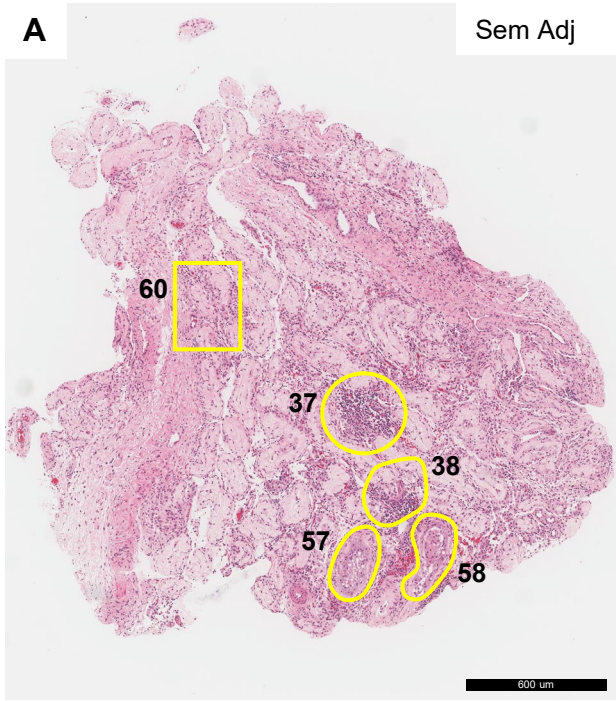**B**

Sem Tum A

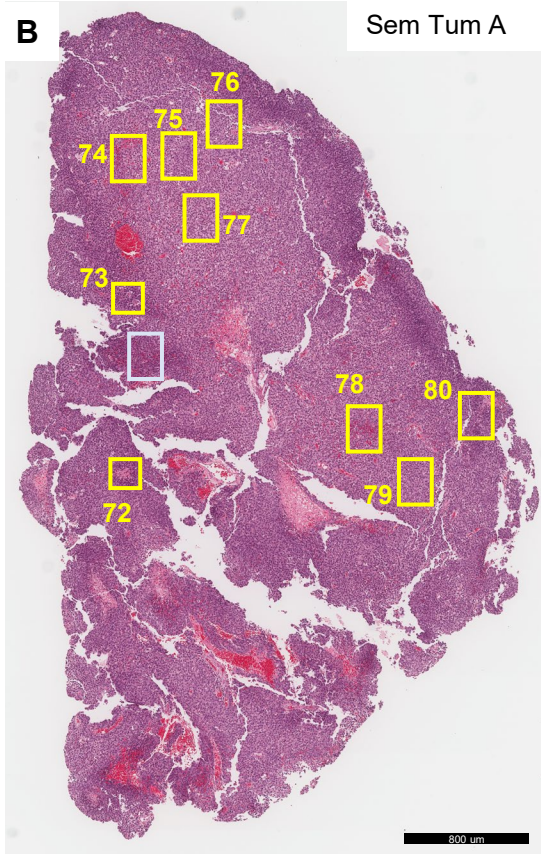**C**

Sem Tum B

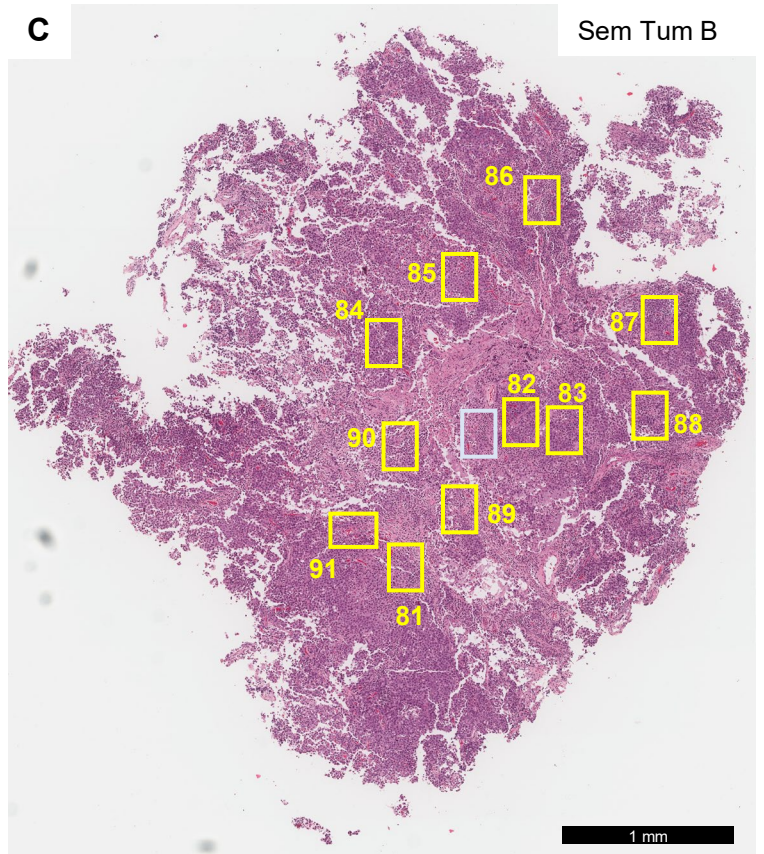

Supplement: Supplementary file 16 — Supporting Information Figure 6 [file ANDR-14-210-s015.pdf]

**A**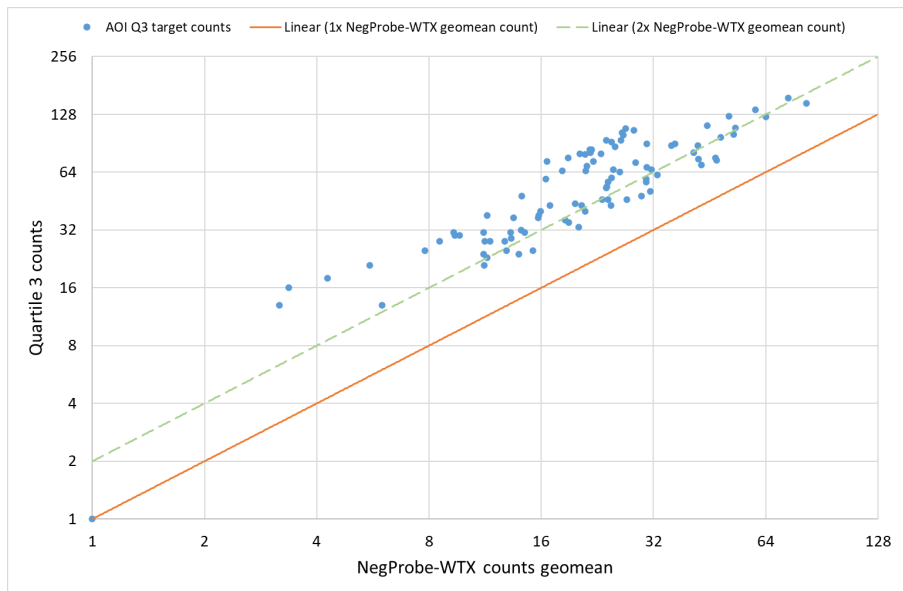**B**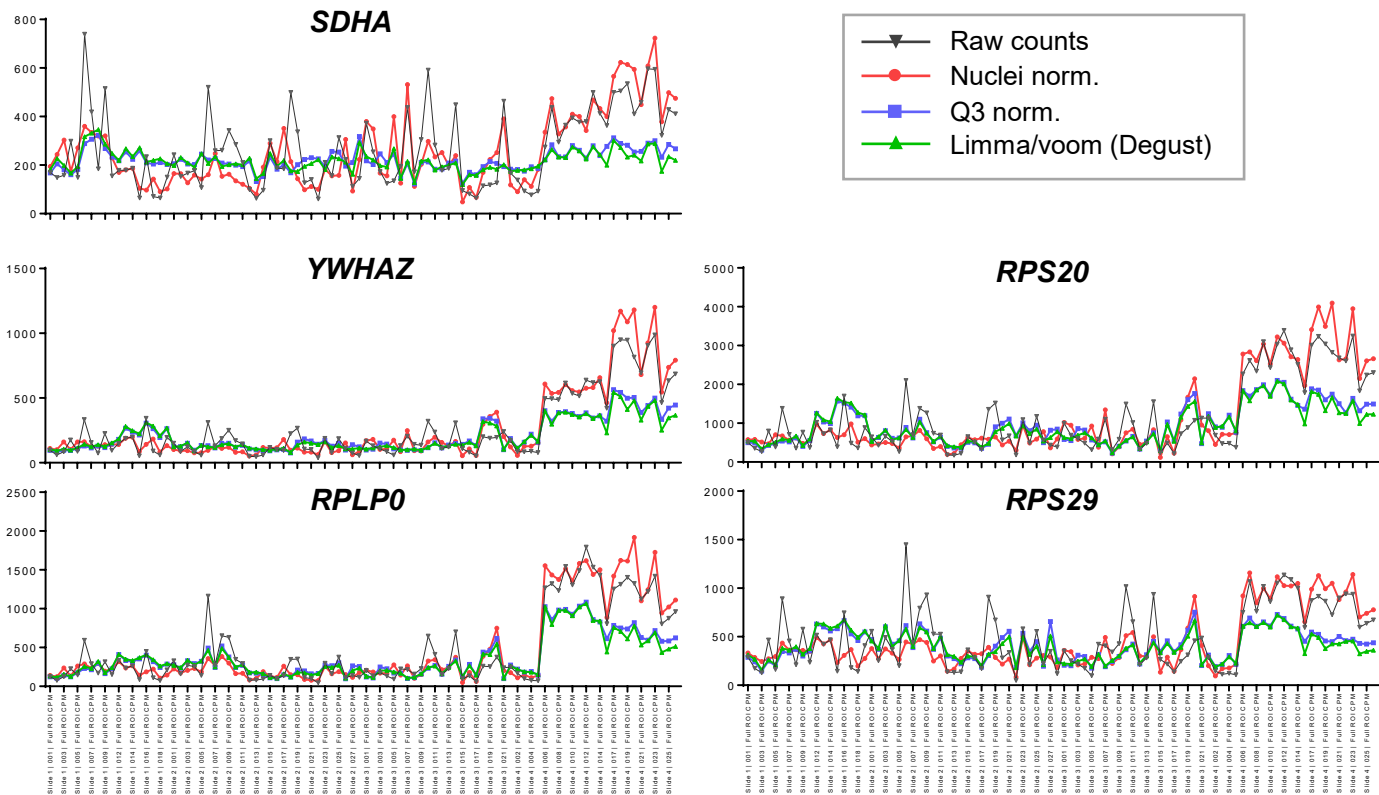

Supplement: Supplementary file 17 — Supporting Information Figure 7 [file ANDR-14-210-s004.pdf]
