# Supplementary material for: Spatial transcriptomics mapping of immune cell and TGFβ signalling pathway heterogeneity in testicular germ cell tumours
Source: Andrology. 2025 Jul 22;14(1):210–27. doi: 10.1111/andr.70100 (PMC12670472; doi:10.1111/andr.70100)
Supplement: Supplementary file 18 — Supporting Information Figure 8 [file ANDR-14-210-s008.pdf]

A

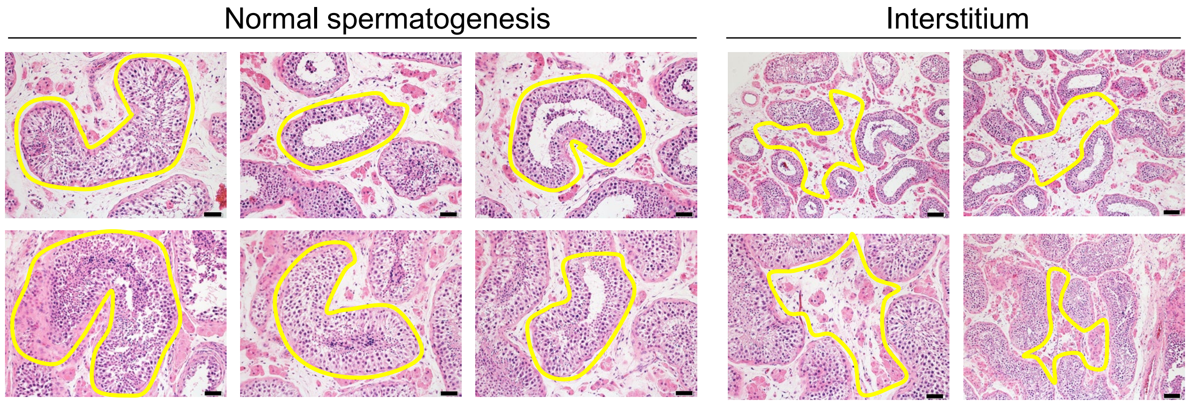

B

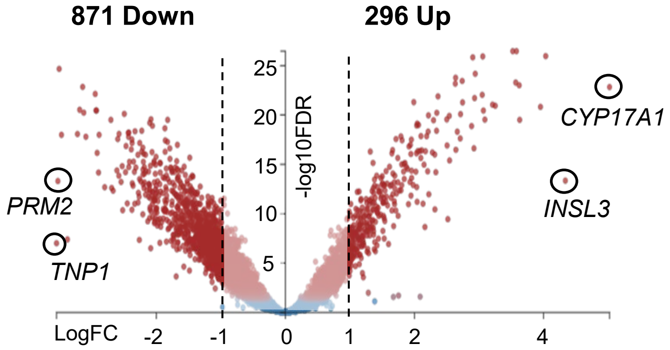

C

DAVID analysis

Upregulated transcripts in interstitium vs NSP	Enrichment score
Steroid, lipid and cholesterol biosynthesis and metabolism	14.75
Extracellular space/region	12.13
Innate immunity	5.43
**Downregulated transcripts in interstitium vs NSP**	**Enrichment score**
Spermatogenesis, differentiation	20.87
Cilium, flagellum, cell projection	19.34
Nucleosome core, chromosome, histone, DN binding	11.48
